# Modified Maddocks Questions Tool in Spanish: evaluating comprehension among child soccer players aged 4–8

**DOI:** 10.3389/fspor.2026.1793910

**Published:** 2026-04-21

**Authors:** Federico Baltar, Camila Blanco, Ailin Collazo, Agustín Figueira, Juliana Meyer, Gabriela Sosa, Florencia Pérez-Vidarte, Santiago Mansilla, Pedro Piccardo, Alcy R. Torres

**Affiliations:** 1 Unidad Académica de Neuropediatría, Universidad de la República, Montevideo, Uruguay; 2 Ciclo Básico Metodológico, Facultad de Medicina, Universidad de la República, Montevideo, Uruguay; 3 Unidad Académica de Métodos Cuantitativos, Facultad de Medicina, Universidad de la República, Montevideo, Uruguay; 4 Department of Pediatrics, Division of Pediatric Neurology, Boston Medical Center, Boston University Chobanian & Avedisian School of Medicine, Boston, MA, United States

**Keywords:** 4–8 years, child soccer players, comprehension, Modified Maddocks Questions Tool, Spanish

## Abstract

**Introduction:**

The Modified Maddocks Questions Tool (MMQT) is part of the Concussion Recognition Protocol used for sideline assessment of suspected concussion. Although widely used in English-speaking populations, it has not been validated in Spanish-speaking children. This study aimed to evaluate comprehension and response accuracy of a Spanish-adapted MMQT in soccer players aged 4–8 years.

**Methods:**

We conducted a cross-sectional observational study in Montevideo, Uruguay, between July and October 2024. Eighty-eight male soccer players aged 4–8 years were assessed at halftime using a Spanish-adapted version of the MMQT. Comprehension was evaluated by asking participants to reformulate each question. Associations between age, comprehension, and response accuracy were analyzed using chi-square or Fisher's exact tests and logistic regression models.

**Results:**

No statistically significant association was found between age and either comprehension or accuracy. Overall comprehension exceeded 96%. However, Question 4 (“Which team won the last match?”) consistently showed a lower correct response rate across all ages. Our findings reinforce previous observations showing that questions assessing recall of past events can be challenging for younger children and suggest caution when evaluating MMQT responses in this age group

**Conclusions:**

The Spanish-adapted MMQT demonstrates high comprehension and may be suitable for sideline concussion screening in children aged 5–8 years. However, Question 4 appears less reliable and should be reconsidered in future versions. A conservative approach is recommended, whereby any incorrect response warrants removal from play for further evaluation.

## 1 Introduction

Soccer is one of the most popular sports worldwide and is a contact sport in which players are at risk of head injuries and concussions (1, 2). The reported incidence of concussions is 0.27–0.41 per 1,000 athletic exposures for school-aged girls and 0.17–0.19 per 1,000 athletic exposures for school-aged boys (3, 4). Head injuries can result from contact between the head and another player's head or body parts, the ground, a goal post, or head-to-ball contact (1, 2, 5). A concussion is a traumatic brain injury caused by a direct blow to the head, neck, or another part of the body (6).

A concussive injury triggers a cascade of molecular alterations, including elevated neurotransmitter release, altered ion flux, hyperglycolysis, and various other metabolic shifts. These complex metabolic disturbances can result in either transient or prolonged neurological deficits. Consequently, concussions must be identified immediately to prevent subsequent injury and mitigate the risk of persistent symptoms (7).

Any athlete suspected of sustaining a concussion must be immediately (on field) evaluated using a standardized tool i.e., CFRP which includes validated modified Maddock's questions to explore orientation and memory. Furthermore, the injured player should be withdrawn from play, monitored for worsening effects, and be encouraged to rest until asymptomatic (6–9). Any athlete suspected of sustaining a concussion must undergo an immediate on-field evaluation using a standardized tool, such as the Concussion Fast Recognition Protocol (CFRP). This protocol includes validated Modified Maddocks Questions (MMQ) designed to assess orientation and memory. Furthermore, the injured player must be withdrawn from play, monitored for any worsening of symptoms, and encouraged to rest until they are completely asymptomatic (6–9).

Based on the preceding data, the CFRP was utilized for the current study. However, it is important to note that the CFRP is not sport-specific and has not yet been validated in Spanish-speaking populations. While the CFRP has previously been used to identify concussions in soccer players, there are currently no data to validate its application in children (7–9). Furthermore, the Maddocks questions are primarily intended for the on-field evaluation of athletes aged 12 years and older who do not present with obvious signs of concussion (6).

The primary objective of this study is to determine the minimum age at which the Modified Maddocks Questions (MMQ) can be reliably applied to soccer players, facilitating the future validation of the Concussion Fast Recognition Protocol (CFRP) in children. We hypothesized that 1) Both comprehension and response accuracy would increase with age across the 4–8-year range. 2) Questions involving the recall of past events would yield lower accuracy compared to orientation-based questions, even among non-concussed children.

## 2 Methods

We conducted an observational, descriptive, cross-sectional study among youth soccer players in Montevideo, Uruguay, using an adapted version of the Modified Maddocks Questions Tool (MMQT). The study took place between July and October 2024. Following authorization from the Secretaria Nacional de Deporte and approval from the respective club academies, participating teams were selected via random sampling. All participants remained anonymous throughout the process.

### 2.1 Participant selection and criteria

Athletes were eligible if they were between the ages of 4 and 8, reflecting the early entry age for organized soccer in Uruguay (10). Exclusion criteria consisted of:

1. Pre-existing neurocognitive deficits.
2. Suspected or confirmed concussion sustained during the match prior to testing.
3. Lack of parental/guardian informed consent or player assent.

### 2.2 Data collection and administration

The MMQT was administered orally by trained investigators during the halftime break to evaluate baseline cognitive function, attention, and comprehension in a non-injured population. To ensure objectivity, the investigators maintained no prior relationship with the participating clubs. Each assessment lasted between 1 and 3 min. If a child provided an incorrect response, the question was reformulated to further assess their capacity for comprehension vs. simple misinterpretation.

Data, including gender, age, and club affiliation, were recorded in an electronic spreadsheet. Responses to each of the five MMQT questions were classified as either correct or incorrect.

### 2.3 Statistical analysis

**Figure 1:**
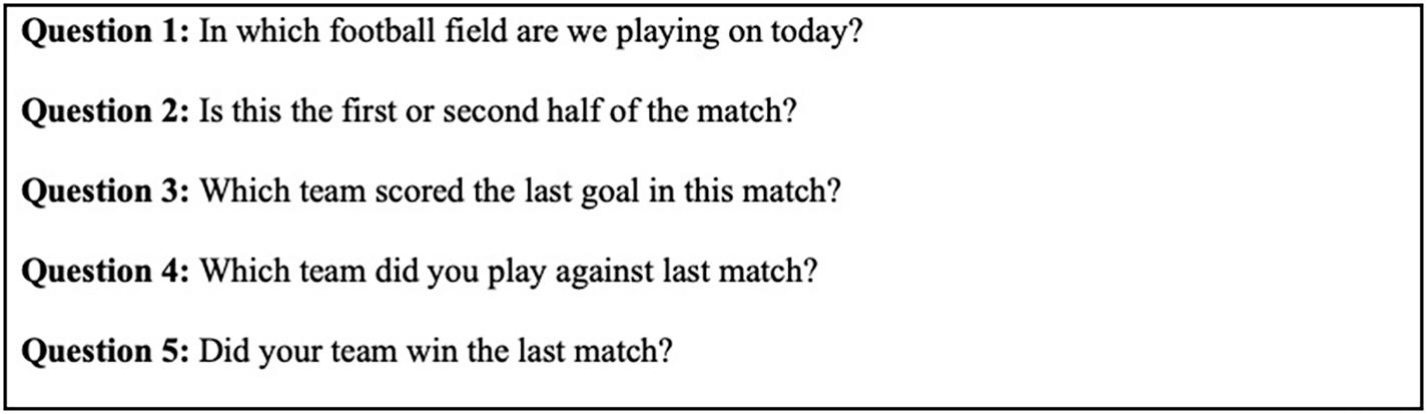
The Modified Maddocks Questions Tool (MMQT).

For data processing, participants were stratified into one-year age intervals. For each age group, we calculated the proportion of correct vs. incorrect responses and evaluated the overall comprehension levels for each of the five specific MMQT items (Figure 1).

### 2.4 Ethical statement

The study protocol and informed consent procedures were approved by the Research Ethics Committee of the School of Medicine at the University of the Republic, Uruguay.

### 2.5 Patient and public involvement

Given the study design, patient and public involvement (PPI) was not required during the planning or execution phases of this research.

### 2.6 Statistical analysis

To examine the association between age and both MMQT response accuracy (correct vs. incorrect) and question comprehension (understood vs. not understood), we employed either the Chi-square (*χ*2) test or Fisher's exact test, as appropriate. In cases where significant associations were detected, *post-hoc* pairwise comparisons of proportions were performed. To account for multiple comparisons and reduce the risk of Type I errors, *p*-values were adjusted using the Bonferroni correction (11).

We also evaluated the association between age and the MMQT variables using logistic regression models, with age as the predictor and each question as a separate binary dependent variable. To estimate the probability of false negative or false positive outcomes based on the number of incorrect responses, we employed a binomial distribution. This calculation assumed independence between responses and utilized the mean probability of an incorrect answer across the questionnaire.

The internal consistency of the MMQT was evaluated using Cronbach's alpha. In doing so, we acknowledged that the tool was originally developed as a brief clinical screening instrument rather than a unidimensional psychometric scale.

Given the exploratory and descriptive nature of this study, no formal *a priori* sample size calculation was performed. Instead, the sample size was determined pragmatically based on participant availability and feasibility during the study period. The final cohort (*n* = 88) was deemed sufficient to estimate comprehension and response accuracy with acceptable precision across the target age range. With this sample, proportions near 90% could be estimated with a margin of error of approximately ±6%–7%. Additionally, this sample size provided approximately 80% power to detect a moderate effect size (Cramer's V ≈ 0,30) in the chi-square analyses.

Statistical analyses were performed using JASP (version 0.18.3). For all tests, a *p*-value of < 0.05 was considered statistically significant.

## 3 Results

The study involved 88 males, aged 4 to 8 years, from six different youth soccer clubs. The distribution of children by age was as follows: 1 child aged 4, 9 children aged 5, 23 children aged 6, 33 children aged 7, and 22 children aged 8. Although all youth soccer clubs in Montevideo accept both sexes, within the age ranges chosen for the study, none of the randomly selected teams had girls on their roster.

Table 1 summarizes the proportion of children who correctly answered and understood each Modified Maddocks Question (MMQ) by age group. Fisher's exact test revealed no statistically significant association between age and either the accuracy of responses or comprehension of questions. Similar findings were obtained using logistic regression models with age as a predictor. However, results should be interpreted cautiously due to the small number of participants in the 4- and 5-year-old groups. When considering the total number of correct responses (range 0-5), the Spearman correlation between age and total score was 0,193 (95% CI: −0,017; 0.387), indicating no association. Conclusions cannot be generalized to 4-year-olds based on the single participant in that group.

**Table 1:** Association between the frequency of correct responses and language comprehension with respect to age.

Question 1	*N*	Respond correctly		*P*-value	Understands		*P*-value
		*N*	%	0.095	*n*	%	1.000
4 years	1	1	100		1	100	
5 years	9	9	100		9	100	
6 years	23	18	78.3		22	95.6	
7 years	33	31	93.9		32	97	
8 years	22	22	100		22	100	
Total	88	81	92		86	97.7	
**Question 2**				0.247			0.737
4 years	1	1	100		1	100	
5 years	9	8	88.9		9	100	
6 years	23	17	73.9		23	100	
7 years	33	30	90.9		31	94	
8 years	22	21	95.5		21	95.5	
Total	88	77	87.5		85	96.6	
**Question 3**				0.362			0.612
4 years	1	1	100		1	100	
5 years	9	9	100		9	100	
6 years	23	17	73.9		23	100	
7 years	33	28	84.8		31	94	
8 years	22	16	72.7		22	100	
Total	88	71	80.7		86	97.7	
**Question 4**				0.111			0.215
4 years	1	0	0		1	100	
5 years	9	6	66.7		8	88.8	
6 years	23	10	43.5		23	100	
7 years	33	19	57.6		31	94	
8 years	22	17	77.3		22	100	
Total	88	52	59.1		85	96.6	
**Question 5**				0.084			1.000
4 years	1	0	0		1	100	
5 years	9	8	88.9		9	100	
6 years	23	17	74		23	100	
7 years	33	28	84.8		32	97	
8 years	22	21	95.4		22	100	
Total	88	74	84.1		87	98.9	

*P*-values correspond to the Fisher’s exact test between age and response or language comprehension.

Not all participants answered every question correctly (Table 2); rather, question 4, showed significantly different proportion of correct responses. In contrast, comprehension rates were similar across all questions.

**Table 2:** Interview results (*n* = 88).

Question	Respond correctly		*p* value	Understands the questions		*p* value
	*n*	%		*n*	%	
1	81	92	**< 0.001**	86	97.7	**0**.**955**
2	77	87.5		85	96.6	
3	71	80.7		86	97.7	
4	52	59.1		85	96.6	
5	74	84.1		87	98.9	

*P*-values corresponding to the Fisheŕs exact test between age and response or language comprehension.

The proportion of correct responses to question 4 was significantly lower compared to the other questions (Table 3). This suggests it may be more difficult or less clearly understood.

**Table 3:** Comparison of the proportion of correct responses among questions (*n* = 88).

Fisher *p* < 0,001	Question 1	Question 2	Question 3	Question 4	Question 5
Question 1		0.995	0.028	<0.001*	0.103
Question 2			0.215	<0.001*	0.516
Question 3				0.002*	0.555
Question 4					<0.001*
Question 5					

*P*-values correspond to the comparison of proportions of correct responses among questions.

*p < 0.005 (Bonferroni correction).

Internal consistency of the five MMQT items was explored using Cronbach's alpha. The overall alpha coefficient was 0,668 (95% CI: 0.556; 0.779), indicating moderate internal consistency. When Question 4 was excluded, Cronbach's alpha decreased to 0,561, suggesting that this item did not reduce overall scale coherence. It should be noted that the MMQ is intended as a brief clinical screening tool rather than a unidimensional psychometric scale; therefore, internal consistency estimates should be interpreted cautiously.

The analysis performed in Table 3 for the comparison of correct responses was not carried out for language comprehension, as no association was found between the two variables (*p* = 0.955, Table 2).

To evaluate the potential for diagnostic error based on the number of incorrect answers, we used the binomial distribution, if each question has the same probability of being answered incorrectly under normal (non-concussed) conditions. Based on responses to Questions 1, 2, 3, and 5, the estimated probability of an incorrect answer was 0.193. Table 4 shows the probability of each of incorrect answers and its corresponding estimated diagnostic error frequency.

**Table 4:** Probability and error frequency based on the number of incorrect responses.

Number of incorrect responses					
	0	1	2	3	4
Probability	0.4237	0.4058	0.1458	0.0233	0.0014
Decision	continue	remove	remove	remove	remove
Error frequency	≈ 1 in every 1.7 times	≈ 1 in every 2.5 times	≈ 1 in every 7 times	≈ 1 in every 43 times	≈ 1 in every 714 times

Calculated from the binomial distribution, using *p* as the average probability of incorrect responses from questions 1, 2, 3, and 5: 0.1932.

For example, if a player answers all four questions correctly, the probability of a false negative (i.e., allowing a concussed player to continue) is 42.4%. Conversely, if a child is removed from the field after just one incorrect answer, the false positive rate (i.e., removing a healthy player) is 40.6%. This rate drops significantly as the number of incorrect responses increases.

This analysis excluded Question 4 due to its significantly lower correct response rate, which could reflect factors unrelated to cognitive status.

*Excluding question 4,* If the child answered all questions correctly, the probability of continuing to play despite having sustained a concussion (false negative) is 1 in 1.7.

If the child is removed from the field after one incorrect answer, the probability of error (removing the child without a concussion) is 1 in 2.5. The probability of error decreases significantly if the child provides an increasing number of incorrect answers (Table 4).

## 4 Discussion

Reports of sports-related concussions, particularly in soccer, have increased in prevalence over the last decade. This trend is likely driven by enhanced diagnostic surveillance, heightened public awareness, and targeted education for parents, coaches, and athletes. Furthermore, the rising competitiveness of youth soccer has contributed to higher injury rates (2). Among school-aged children, it is estimated that over 50% of concussions occur during recreational play (7). In most competitive settings, certified athletic trainers serve as the primary responders for the initial assessment and management of these injuries.

In recreational sports settings, however, it is essential that parents and coaches possess the necessary knowledge to recognize concussion symptoms (2). The Concussion Fast Recognition Protocol (CFRP), implemented in 2014, was originally designed for adult athletes; consequently, there remains a lack of data regarding its utility and efficacy in pediatric populations (9).

Our findings indicate no significant association between the three primary parameters investigated: age, auditory comprehension, and the frequency of correct responses.

Notably, most of the study population consisted of children aged 6, 7, and 8 years, with a smaller representation of younger participants (one 4-year-old and nine 5-year-olds). Crucially, no statistically significant differences in performance were observed among the children in the 6–8 age range.

Across all age groups, participants demonstrated a high degree of language comprehension (≥96%), providing correct answers in most instances. However, Question 4 consistently yielded incorrect responses across the cohort. This finding suggests that Question 4 may be unsuitable for this specific age group; we recommend its exclusion from the protocol should further studies confirm these preliminary results. Consequently, any child failing to provide a correct response to the remaining items should be managed with a high degree of clinical suspicion and, as a precautionary measure, removed from play for formal medical assessment and rest.

The Maddocks questions were originally developed for professional Australian rules football players. The initial 14-item tool assessed orientation and recent memory and was typically administered within 10 min of a suspected concussion or upon the patient regaining consciousness (8, 12, 13). Historically, and consistent with our observations regarding Question 4, items evaluating recent memory have proven more sensitive in distinguishing between concussed and non-concussed athletes, whereas questions assessing remote events often proved challenging for all players, regardless of injury status (13, 14).

To the best of our knowledge, there are no published reports on the Spanish validation of the Modified Maddocks Questions Tool (MMQT) for young children, although it is a recognized instrument for differentiating between concussed and non-concussed adolescents and adults during the acute phase of injury. Since its inception in 2005, the Sport Concussion Assessment Tool (SCAT) has undergone several revisions, consistently including the Maddocks questions as a core component (15). However, research has highlighted significant performance variations linked to demographic variables, as well as cultural and linguistic differences (8, 15, 16).

While the SCAT is a standardized tool with universal applicability across disciplines including soccer, hockey, basketball, and American football, its development and validation have been most robustly established within rugby (16–23). It is therefore critical to recognize that a literal translation of the SCAT may be inadequate; cultural and linguistic adaptations are essential to ensure effective communication and prevent clinical misunderstandings (8).

The impact of language on concussion evaluation in youth athletes remains poorly understood (14). Studies utilizing the Child SCAT5 have found that Spanish-speaking children even when evaluated in English reported fewer symptoms and performed worse on specific subtests compared to their English-speaking peers. These discrepancies may be attributed to social determinants of health, such as socioeconomic status and acculturation, or inherent psychometric limitations of the tool. This underscores the need for pediatric concussion instruments to account for cultural and linguistic nuances to ensure diagnostic validity (16). Notably, while the MMQT has been validated in Chinese, Arabic, and Italian within various SCAT versions, a Spanish pediatric validation remains a critical gap (24–26).

Athletic trainers and specialized healthcare personnel are in short supply in child sports. Therefore, it is necessary to raise awareness and provide training on the use of CRT6 to all adults supervising child/adolescent sports. The CRT6 created by the Concussion in Sport Group (CISG) has been translated into several languages (16).

This study supports the utility of a Spanish-adapted MMQT for concussion screening in children aged 5–8 years. Comprehension was consistently high across age groups, although the ability to answer correctly was variable especially for questions involving recall of past events (e.g., Question 4). Previous studies on the original Maddock questions have similarly reported that items involving recall of past events are less discriminative and more variable, even among older athletes (13). In contrast, orientation-based questions (e.g., field, half of match) rely on immediate contextual awareness and appear more robust across age groups. Given the lower performance on Question 4 despite high comprehension, its exclusion from the MMQT for this age group is recommended.

While the study was limited to male athletes and had limited representation among 4- and 5-year-olds, the results are promising. Future research should expand validation to girls, younger children, and other sports.

Given the limited availability of trained medical staff at youth sports events, tools like MMQT must be easily understood by non-medical adults (e.g., parents, coaches) and appropriately adapted to cultural and linguistic contexts.

### 4.1 Clinical implications

Identifying concussions in the pediatric population remains a formidable diagnostic challenge, largely due to the communication barriers inherent to early cognitive development and a paucity of validated assessment tools. In this context, the present study offers novel and clinically significant evidence by demonstrating the feasibility of the Modified Maddocks Questions Tool (MMQT) for use among Spanish-speaking children aged 5 to 8 years.

### 4.2 Limitations

This study is subject to several limitations. First, the sample consisted exclusively of male participants, as the randomly selected teams within the target age range lacked female players; consequently, these findings may not be generalizable to female athletes. Second, the younger cohorts were underrepresented comprising only one 4-year-old and nine 5-year-olds which limits the strength of the conclusions for these specific age groups. Third, the relatively small, convenience-based sample size may have reduced statistical power, particularly for subgroup analyses.

Furthermore, the cross-sectional design and administration of the MMQT in a non-concussed setting during halftime may not fully capture the ecological validity of actual sideline conditions following a head injury. The practice of reformulating incorrect answers to assess comprehension, while helpful, may have introduced a degree of researcher subjectivity. Finally, as the MMQT has not been formally validated in Spanish-speaking pediatric populations, cultural or contextual factors may further limit its broader application. Future research incorporating larger, more diverse cohorts is warranted.

## 5 Conclusions

Modified Maddocks Questions Tool (MMQT) is a feasible sideline concussion screening instrument for Spanish-speaking male athletes aged 5 to 8 years. However, due to its consistently lower accuracy in this demographic, we recommend that Question 4 be excluded from routine use. Clinically, any child who provides one or more incorrect responses should be immediately removed from play and referred for a comprehensive medical evaluation.

## Data Availability

The raw data supporting the conclusions of this article will be made available by the authors, without undue reservation.
